# Meta‐analysis shows that environmental DNA outperforms traditional surveys, but warrants better reporting standards

**DOI:** 10.1002/ece3.7382

**Published:** 2021-03-18

**Authors:** Julija Fediajevaite, Victoria Priestley, Richard Arnold, Vincent Savolainen

**Affiliations:** ^1^ Department of Life Sciences Imperial College London London UK; ^2^ Thomson Environmental Consultants Compass House Surrey Research Park Guildford UK

**Keywords:** ecological survey, environmental DNA, meta‐analysis, probability of detection, species detection, traditional methods

## Abstract

1. Decades of environmental DNA (eDNA) method application, spanning a wide variety of taxa and habitats, has advanced our understanding of eDNA and underlined its value as a tool for conservation practitioners. The general consensus is that eDNA methods are more accurate and cost‐effective than traditional survey methods. However, they are formally approved for just a few species globally (e.g., Bighead Carp, Silver Carp, Great Crested Newt). We conducted a meta‐analysis of studies that directly compare eDNA with traditional surveys to evaluate the assertion that eDNA methods are consistently “better.”
2. Environmental DNA publications for multiple species or single macro‐organism detection were identified using the Web of Science, by searching “eDNA” and “environmental DNA” across papers published between 1970 and 2020. The methods used, focal taxa, habitats surveyed, and quantitative and categorical results were collated and analyzed to determine whether and under what circumstances eDNA outperforms traditional surveys.
3. Results show that eDNA methods are cheaper, more sensitive, and detect more species than traditional methods. This is, however, taxa‐dependent, with amphibians having the highest potential for detection by eDNA survey. Perhaps most strikingly, of the 535 papers reviewed just 49 quantified the probability of detection for both eDNA and traditional survey methods and studies were three times more likely to give qualitative statements of performance.
4. *Synthesis and applications*: The results of this meta‐analysis demonstrate that where there is a direct comparison, eDNA surveys of macro‐organisms are more accurate and efficient than traditional surveys. This conclusion, however, is based on just a fraction of available eDNA papers as most do not offer this granularity. We recommend that conclusions are substantiated with comparable and quantitative data. Where a direct comparison has not been made, we caution against the use of qualitative statements about relative performance. This consistency and rigor will simplify how the eDNA research community tracks methods‐based advances and will also provide greater clarity for conservation practitioners. To this end suggest reporting standards for eDNA studies.

## Graphical abstract

A meta‐analysis shows that environmental DNA outperforms traditional methods for biodiversity monitoring, both in terms of cost and efficiency.

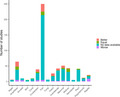

## 1 INTRODUCTION

Environmental DNA (eDNA) is genetic material extracted from environmental samples. It can be used to infer the presence of single or multiple species (metabarcoding) and estimate population abundance and density (Doi, Takahara, et al., 2015; Doi, Uchii, et al., 2015; Dunn et al., 2017; Evans et al., 2016; Knudsen et al., 2019; Tillotson et al., 2018). The application of eDNA for ecology and conservation research has increased at an exponential rate over the last 20 years (Jiang & Yang, 2017), with more than 50 papers published year on year since 2016 (Beng & Corlett, 2020), from eDNA use for the detection of zooplankton (Yang & Zhang, 2020) to large mammals (Hauger et al., 2020) and many taxa in between. Creative and diverse sample types, such as salt licks (Ishige et al., 2017), blood meal (Rodgers et al., 2017), snow tracks (Franklin et al., 2019), as well as more conventional sampling of water (Brys et al., 2020), sediment (DiBattista et al., 2019) and soil (Marquina et al., 2019), have been taken from all major types of habitats: terrestrial (Abrams et al., 2019), marine (Closeket al., 2019), estuarine (Siegenthaler et al., 2019), lentic (Parsley et al., 2020), and lotic (Takahara et al., 2019).

The eDNA method has been described as more sensitive (Biggs et al., 2015; Dejean et al., 2012; Fernández et al., 2019; Hinlo et al., 2017; Jerdeet al., 2011; Pilliod et al., 2013; Schneider et al., 2016) and cheaper (Akre et al., 2019; Miya et al., 2015; Stoeckle et al., 2016) than traditional survey methods. Environmental DNA surveys are, therefore, recognized as a powerful tool for monitoring endangered species (Akamatsu et al., 2020; Brozio et al., 2017; Day et al., 2019; Laramie et al., 2015; Schmelzle & Kinziger, 2016; Thomsen et al., 2012) with the advantage of being nondestructive (Grealy et al., 2015; Hunter et al., 2015; Knudsen et al., 2019; Li et al., 2019). Environmental DNA methodologies are also considered less prone to morphological identification bias (Buxton et al., 2018; Li et al., 2019) and spatial autocorrelation (Deiner et al., 2016) than traditional monitoring methods.

Although eDNA methods have many advantages, detection probability is dependent on the life history of target species (Takeuchi et al., 2019), behavior (Dunn et al., 2017), and on population density (Baldigo et al., 2017). Detection of eDNA is also affected by environmental conditions (Harper, Buxton, et al., 2019; Harper, Griffiths, et al., 2019), the presence of polymerase chain reaction (PCR) inhibitors, distance from shedding source (Goldberg et al., 2016), primer degeneration, or variable PCR efficacy (Nester et al., 2020). These factors can all result in inference errors (Bohmann et al., 2014; Casey et al., 2012; Darling & Mahon, 2011; Goldberg et al., 2016) and false–negatives (Cowart et al., 2015; Mauvisseau et al., 2019; Piaggio et al., 2014; Rice et al., 2018).

In contrast to traditional survey methods (Stoeckle et al., 2016), eDNA techniques can also lead to false‐positives (Cowart et al., 2018; Dejean et al., 2012; Ficetola et al., 2015; Gueuning et al., 2019), which might occur due to resuspension of eDNA from sediment (Buxton et al., 2018), transfer of eDNA from its originating environment to a sampling site (i.e., allochthonous eDNA) (Goldberg et al., 2016; Harper, Buxton, et al., 2019; Harper, Griffiths, et al., 2019), or equipment contamination (Bohmann et al., 2014). Compared to traditional methods, eDNA is currently less able to provide complete information about population status and stability (Bailey et al., 2019; Rose et al., 2019; Ulibarri et al., 2017), sex, size, or health condition (Goldberg et al., 2016).

The limitations could explain why eDNA methods are not widely approved to survey biodiversity (Evans et al., 2017), with notable exceptions of priority conservation species Great Crested Newt (*Triturus cristatus* Laurenti) in the UK (Biggs et al., 2015), as well as highly destructive invasive Bighead Carp (*Hypophthalmichthys nobilis* Richardson) and Silver Carp (*Hypophthalmichthys molitrix* Valenciennes) in the United States (Amberget al., 2015).

One way to test the eDNA method's validity is to directly compare it with traditional monitoring methods, as pioneered by Thomsen et al. (2012). Many comparative studies have been conducted (e.g., Fernández et al., 2019; Ficetola et al., 2015; Hinlo et al., 2017; Jo et al., 2020; Pilliod et al., 2013; Rice et al., 2018; Wilcox et al., 2016), encompassing a variety of traditional method types, shown in Table 1. However, there have been few attempts to synthesize the results of comparative analyses.

**TABLE 1:** Types of traditional biodiversity surveys, which have been compared to eDNA method

Capture‐based surveys	Visual search surveys	Acoustic surveys
Angling (O'Sullivan et al., 2020) Baited trapping (Riascos et al., 2018) Blacklight traps (Maslo et al., 2017) Bottle trapping (Cai et al., 2017) Bottom trawling (Thomsen et al., 2012 Cast netting (Fujii et al., 2019) Dip netting (Fujii et al., 2019) Electrofishing (Fernández et al., 2019) Fungi fruiting body collection (Shirouzu et al., 2016) Fyke netting (Harper, Griffiths, et al., 2019) Gill netting (Gillet et al., 2018) Hand picking (Doi et al., 2020) Host necropsy (Trujillo‐González et al., 2019) Kick netting (Rice et al., 2018) Minnow traps (Fujii et al., 2019) Mosquito magnets (Boerlijst et al., 2019) Night aquatic funnel traps (Rose et al., 2019) Pollen analysis (Sjögren et al., 2017) Seine hauls (Johnston & Janosik, 2019) Surber sampling (McInerney & Rees, 2018) Tow netting (Minegishi et al., 2019) Zooplankton netting (Walsh et al., 2019)	Baited remote underwater video station (BRUVS) (Stat et al., 2019) Camera traps (Sales et al., 2020) Diving (Wood et al., 2019) Egg search (Harper et al., 2018) Fossil analysis (Parducci et al., 2019) Scat (Thomsen et al., 2012) Snorkelling (O’Sullivan et al., 2020) Snow tracks (Franklin et al., 2019) Tadpole search (Dejeanet al., 2012) Torchlight surveys (Rees et al., 2014) Underwater visual census (UVC) (Alsoset al., 2018) Water binocular (Trebitz et al., 2019) Visual encounter surveys (VESs) (Schütz et al., 2020)	Audio strip transects (ASTs) (Dejean et al., 2012) Calling surveys (Lopes et al., 2017) Hydroacoustics (Coulter et al., 2018) Telemetry (Mize et al., 2019)

There are several reviews of eDNA research (Beng & Corlett, 2020; Hering et al., 2018; Lamb et al., 2019; McElroy et al., 2020; Yates et al., 2019), but to our knowledge, there has been no attempt to address the question of whether eDNA consistently outperforms traditional methods nor whether that outcome is influenced by the taxon studied, habitat type, or methodology (Buxton et al., 2017; Furlan et al., 2019; Klymus et al., 2015).

Therefore here, we conducted a meta‐analysis of studies that directly compare eDNA with traditional survey methods to (i) determine whether eDNA performs “better” than traditional methods and (ii) to understand what factors influence this outcome.

## 2 MATERIALS AND METHODS

### 2.1 Identifying relevant papers

The database of papers was created by searching the terms “environmental DNA” and “eDNA” using ISI Web of Science in topic (exact search). Search results were limited to English language studies published between 1st January 1970 and 5th May 2020. The search was performed between 22nd April and 5th May 2020.

**FIGURE 1:**
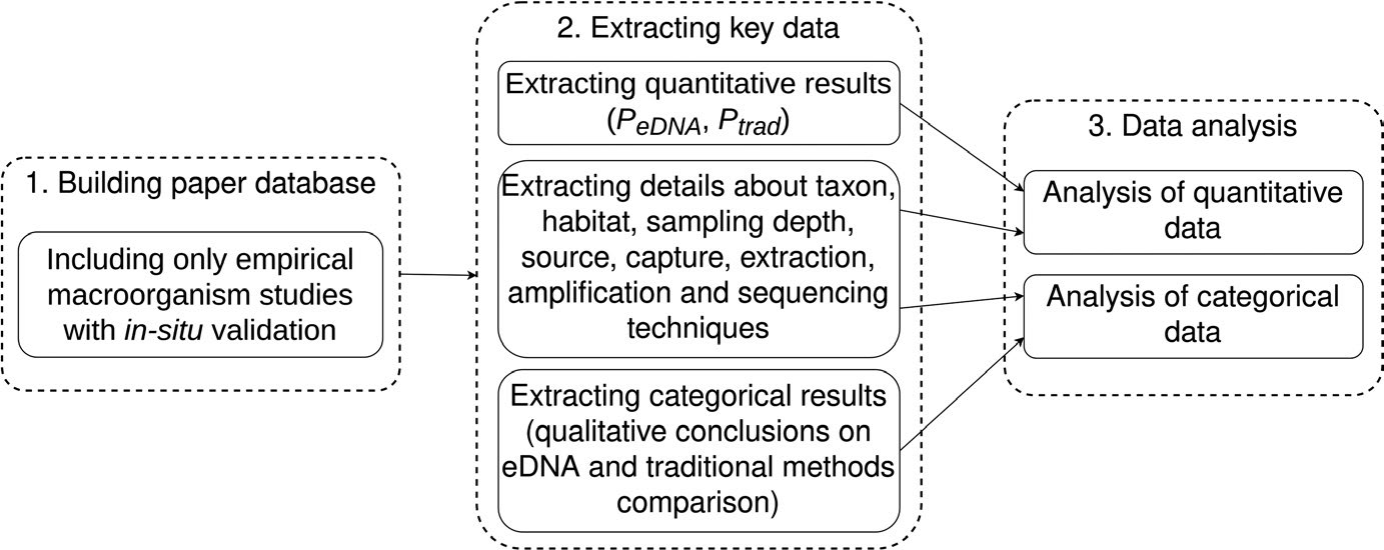
Workflow of this study consisted of three main steps: building paper database, extracting key information from papers, and conducting analysis of quantitative and categorical data

The database was manually refined (Figure 1) by scanning title, abstract, and methods section of the main text to identify and remove papers that were out of scope: microbiological, metabolic, protein‐based detection, nonempirical, and purely technical papers (e.g., development of markers or amplification assays tested ex‐situ).

### 2.2 Extracting key data

Information about the publication year, taxa studied, habitat, method used, and results obtained were extracted from the refined database. Details about the methods, including sampling depth, capture technique, pore size of filter membrane, volume of water filtered, source of eDNA sampled, DNA extraction, amplification, and sequencing techniques and amplification markers, were recorded and are available in Table S1. Two types of result data were collected: quantitative continuous (probability of detection) and categorical (comparison of eDNA and traditional methods outcome).

The probability of detection (*P*_eDNA_ for eDNA probability of detection and *P*_trad_ for traditional methods probability of detection) is a parameter that accounts for environmental stochasticity and imperfect detection (Schmidt & Pellet, 2009) and varies depending on the species as well as the method used, hence it can be utilized as a proxy to infer sensitivity (Schmidt & Pellet, 2009). Across the papers analyzed in this study, *P*_eDNA_ and *P*_trad_ were estimated from the species occupancy models (Dougherty et al., 2016; Rose et al., 2019), where the presence or absence of the species is described as the Bernoulli trial (Schmidt et al., 2013), the N‐mixture models of population abundance (Kéry, 2018), or as a number of positive observations/replicates divided by a total amount of trials/samples collected (Pilliod et al., 2013).

Categorical data for eDNA versus traditional methods were either based on results from the same study (83% of papers), or a comparison made by the authors with historical traditional survey methods (17% of papers). Authors of comparative studies used three criteria: sensitivity, cost‐effectiveness, and number of detectable species. The outcome of the comparison was then assigned to “better,” “equal,” or “worse” for the criteria of sensitivity and number of detectable species; and “cheaper,” “equal,” or “more expensive” for the cost‐effectiveness criterion. In some instances, authors reported that the results of two methods correlated; however, they could not conclude that the methods performed equally well. In such case, the term “correlated” was assigned. If the authors did not provide a clear outcome of the comparison, the term “unclear” was given.

### 2.3 Analysis of quantitative data

The Shapiro‐Wilk test was used for checking the distribution normality of *P*_eDNA_ and *P*_trad_. The comparison of *P*_eDNA_ and *P*_trad_ was performed by the Wilcoxon signed‐rank test for dependent samples.

The influence of abiotic and biotic factors on eDNA versus traditional methods was evaluated by the Kruskal‐Wallis one‐way analysis of variance and the subsequent Conover‐Iman post hoc test among the *P*_eDNA_ of different groups (taxa, habitat, and methods).

To test whether variation in *P*_eDNA_ was explained by filter membrane pore size, the volume of water filtered or the date of publication, a generalized linear model (GLM) was developed, assuming quasi‐binomial error distribution and using link logit canonical function. GLM was used instead of linear model due to *P*_eDNA_ being proportion data, varying from 0 to 1. Quasi‐binomial error distribution was assumed because binomial fitting resulted in under‐dispersion (Equation S1). Pseudo *R* ^2^ parameter (Equation S2) was calculated as in Zuur et al. (2013).

Correlation between volume of water filtered and filter membrane pore size was investigated by Spearman's correlation test.

### 2.4 Analysis of categorical data

For each category of eDNA versus traditional methods comparison outcome (“better,” “equal,” “worse,” “cheaper,” “more expensive,” “correlated,” “unclear”) the number of studies falling into the categories was counted. To test whether the outcome of eDNA and traditional surveys comparison was affected by abiotic and biotic factors, the *χ* ^2^ test of independence was performed for contingency tables of comparison outcomes and different categories of methods, taxa, and habitats. Where *χ* ^2^ test indicated a significant relationship between comparison outcomes and factors tested, a multiple correspondence analysis was subsequently performed to visualize that relationship.

All data analysis were performed using R version 3.6.1. (R Development Core Team, 2019).

## 3 RESULTS

**FIGURE 2:**
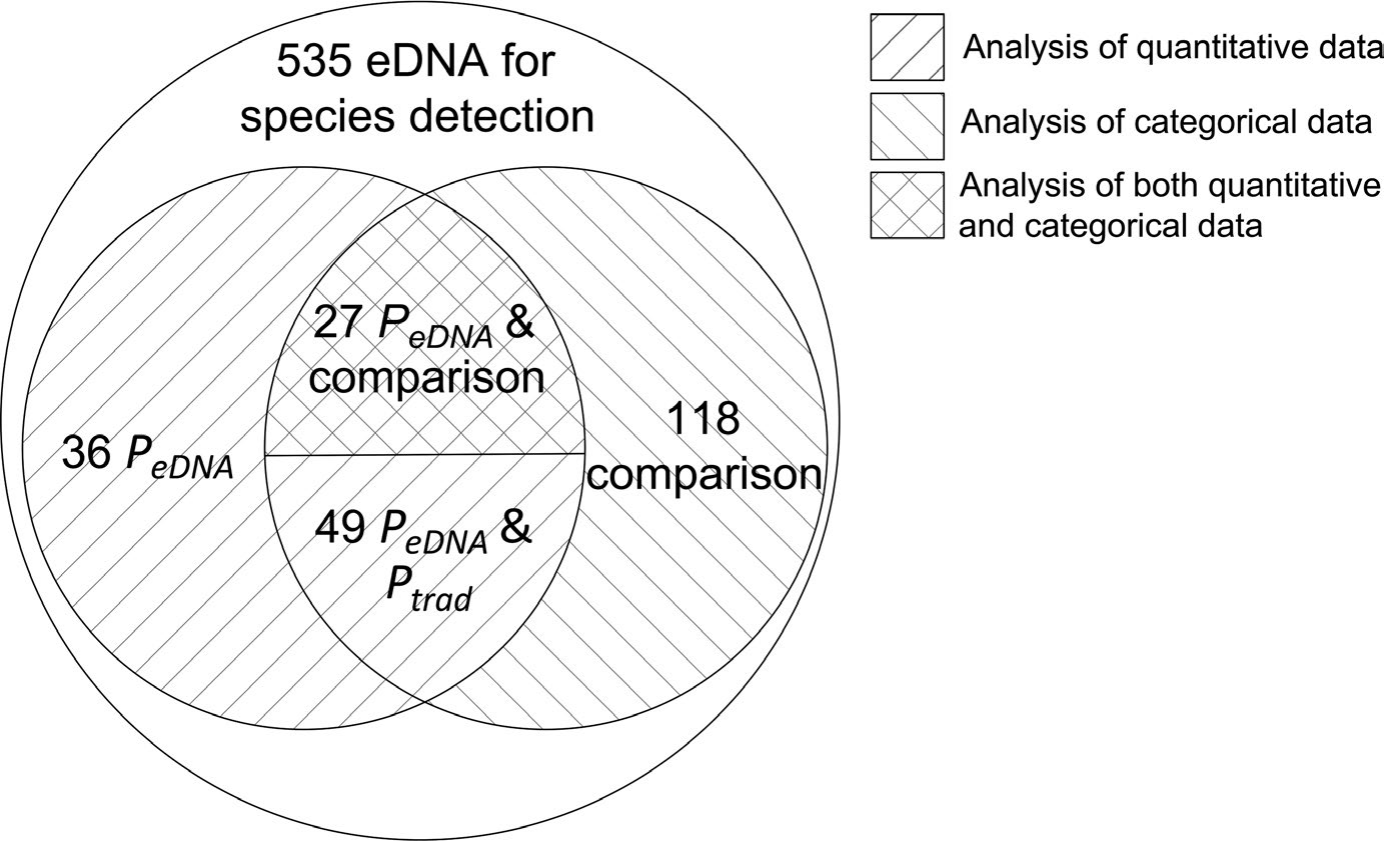
A Venn diagram of different paper classes analysed in this study, based on the type of results they reported. Papers that reported any probability of detection, were used for quantitative data analysis, while those that did not report probability of detection but compared eDNA and traditional methods, were used for categorical analysis. Papers that both reported *P*_eDNA_ and did comparison were used for both quantitative and categorical analysis

A total of 535 papers were identified as providing results of empirical eDNA studies for macroscopic species detection. Out of these 535, 230 papers (43%) report quantitative results (*P*_eDNA_ and/or *P*_trad_) or qualitative results (categories of eDNA performing “better,” “worse,” or “equally well” as traditional methods) that were analyzed in this study. A total of 194 papers (36%) describes a comparison between eDNA and traditional methods (Figure 2). A total of 115 papers gives *P*_eDNA_ results, of which 76 also conduct a comparison of eDNA and traditional methods. Of these, only 49 papers also report the *P*_trad_, meaning that just 9% of the 535 papers could be used for a quantitative comparison of eDNA and traditional method sensitivity.

The 49 studies that provided both *P*_eDNA_ and *P*_trad_ were mainly conducted in freshwater lentic (47%) or lotic (38%) systems and mostly studied fish (25%) and amphibians (25%). These papers estimated the probability of detection by using both eDNA and traditional methods at the same study sites.

### 3.1 Does eDNA perform “better” than traditional methods?

#### 3.1.1 Analysis of quantitative data

**FIGURE 3:**
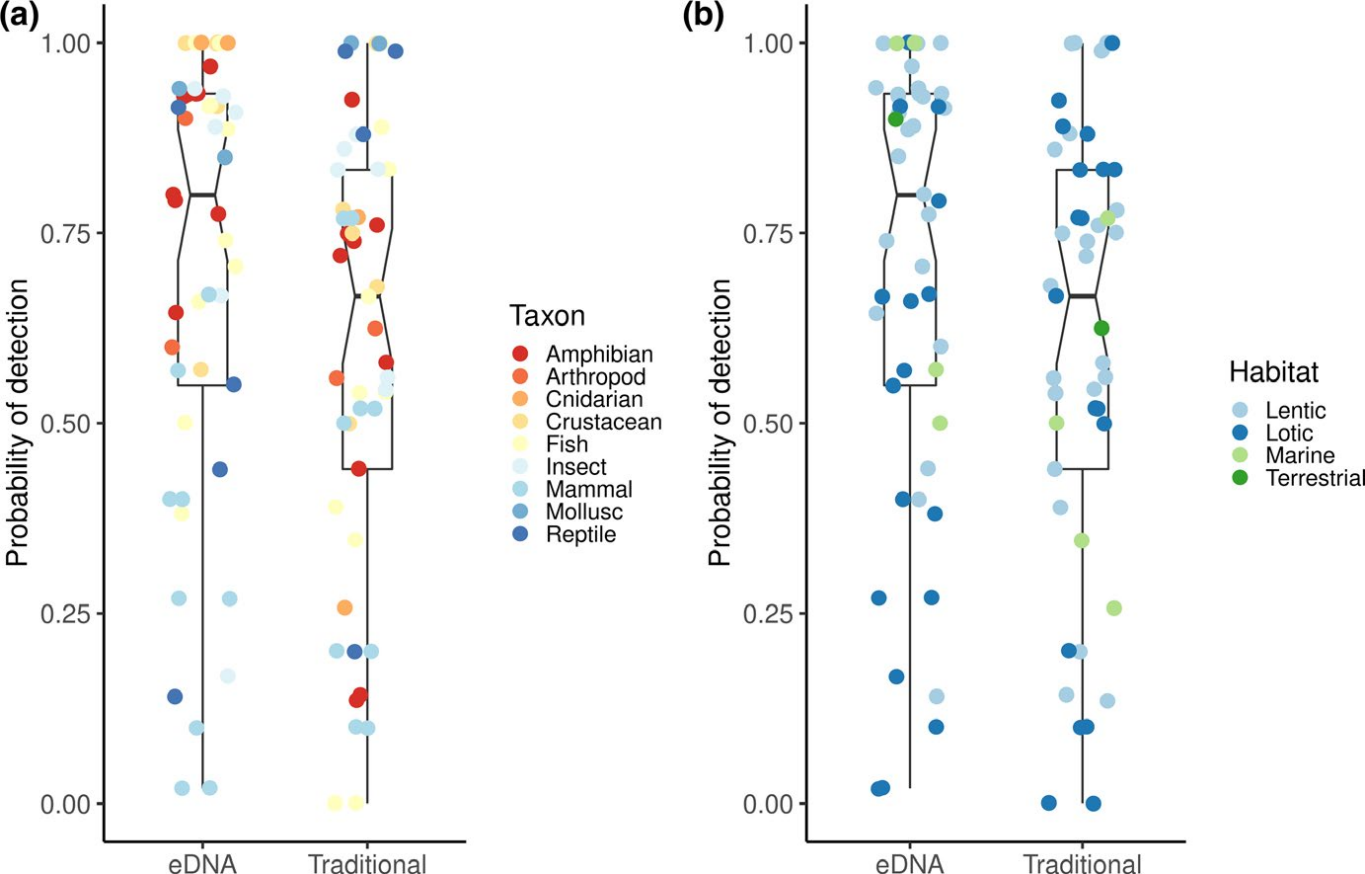
*P*_eDNA_ and *P*_trad_ as reported in 49 studies that compared eDNA and traditional methods at the same study sites for: (a) different habitats and (b) taxa. Notches indicate medians

A collection of all *P*_eDNA_ and *P*_trad_ extracted from the 49 studies mentioned above were not normally distributed, as detected by the Shapiro‐Wilk test (for *P*_eDNA_: *W* = 0.86, *p*‐value < .001, for *P*_trad_: *W* = 0.93, *p*‐value < .01). Wilcoxon's signed‐rank test, for comparing probabilities of detection mean ranks, revealed a significant difference between eDNA and traditional methods (*W* = 1,487, *p* = .04, *n*_eDNA_ = 49, *n*_trad_ = 49), suggesting that *P*_eDNA_ had higher median than *P*_trad_ (Figure 3a,b).

#### 3.1.2 Analysis of categorical data

**FIGURE 4:**
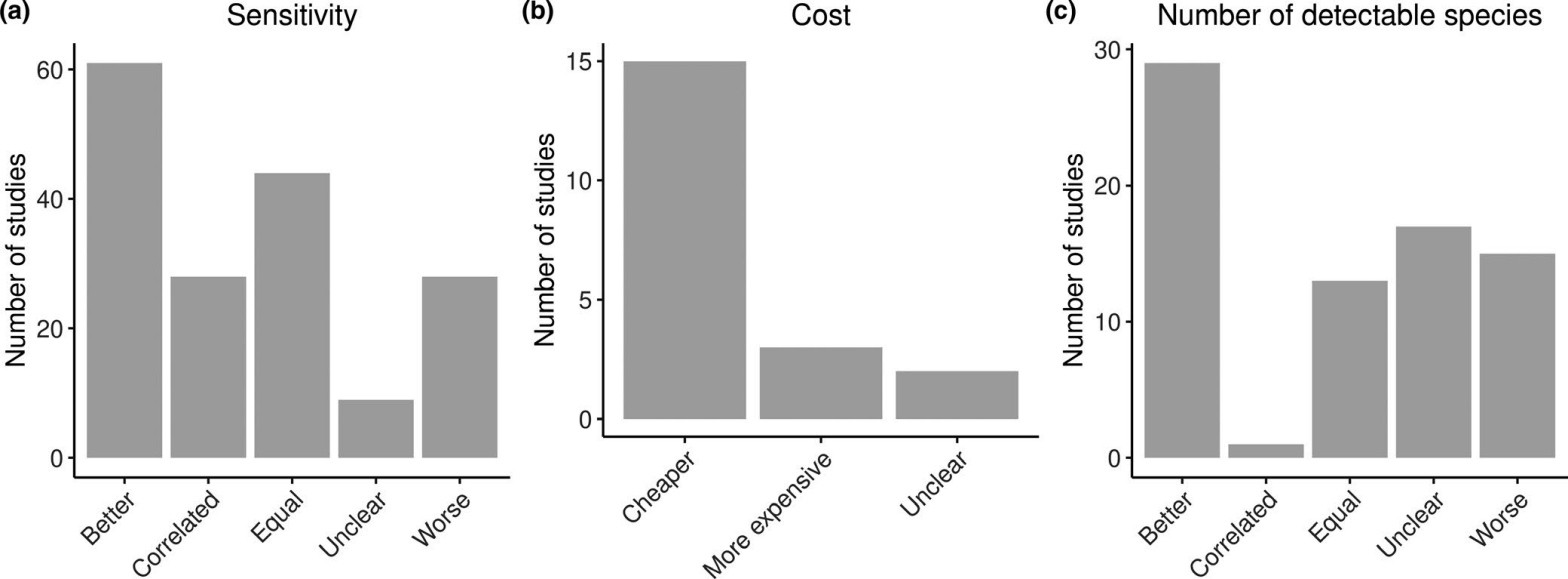
Comparison of eDNA and traditional methods by sensitivity, cost‐efficiency, and number of detectable species, as counted from 194 publications

Of the 194 papers that directly compare eDNA with traditional methods, 170 used sensitivity as the main criterion, 19 focused on cost‐effectiveness, and 75 reported results in terms of the number of detectable species. Across all three criteria, the majority of studies (61 for sensitivity, 15 for cost, and 29 for detectable species) found that eDNA performed better than traditional methods (Figure 4).

### 3.2 What factors influence the comparison of eDNA and traditional methods?

#### 3.2.1 Analysis of quantitative data

Kruskal‐Wallis analysis of variance indicated a significant difference among the *P_eDNA_* values obtained by using different amplification methods (Kruskal‐Wallis *χ* ^2^ = 11.74, *p* = .002, *n* = 150) and by sampling various eDNA sources (Kruskal‐Wallis *χ* ^2^ = 14.45, *p* = .04, *n* = 152). Other factors had no effect on *P*_eDNA_ (Table S2).

**FIGURE 5:**
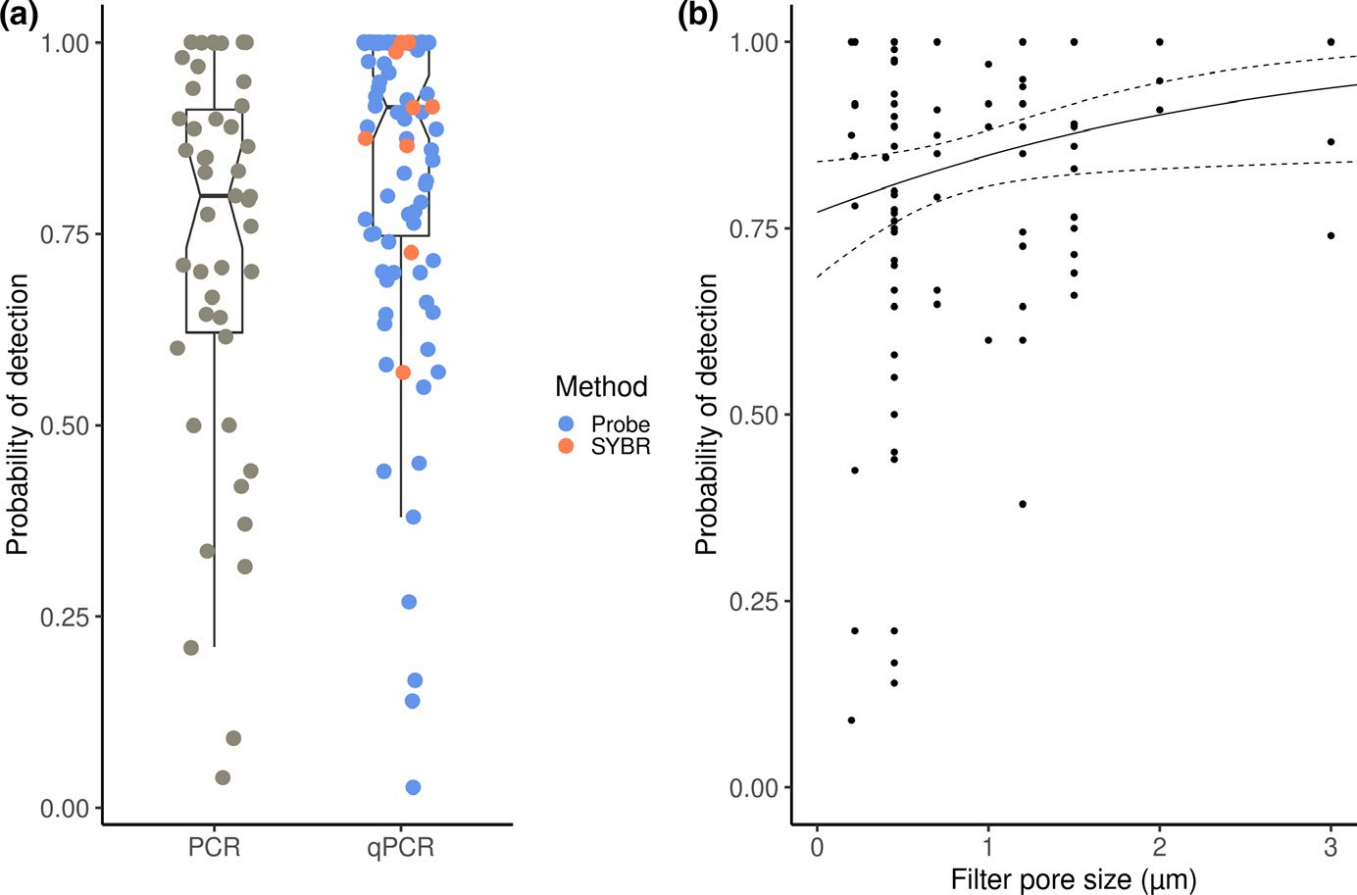
(a) eDNA probabilities of detection by using two different amplification methods—PCR and qPCR (*n*_PCR_ = 46, *n*_qPCR_ = 93). The latter is differentiated into probe‐based qPCR (blue points, *n* = 82) and SYBR‐based qPCR (orange points, *n* = 11). Notches indicate medians. (b) Relationship between *P*_eDNA_ and the filter membrane pore size. Model fit is plotted as a solid line, with 95% confidence level as dashed lines

Subsequent Conover‐Iman post hoc analysis detected a significant difference between quantitative polymerase chain reaction (qPCR) and conventional PCR *P*_eDNA_ values (*z*‐statistic = −2.79, *p* = .02, *n*_PCR_ = 46, *n*_qPCR_ = 93). The test indicated that the qPCR resulted in significantly higher *P*_eDNA_ values than PCR (Figure 5a). Among the qPCR group, 82 studies used probe‐based qPCR, and 11 performed SYBR‐based qPCR. Other amplification types, such as ddPCR and LAMP had insufficient sample sizes (*n* < 10), hence could not be included in the analysis.

Although Kruskal‐Wallis analysis of variance suggested significant differences between different eDNA sample types, the volume of sample used was rarely reported (*n* < 10) (with the exception of water) and it was not possible to perform the Conover‐Iman post hoc analysis.

The effect of filter membrane pore size and volume of water filtered was tested by modeling approach, which suggested a significant positive association (*p* = .04, *n* = 117, Pseudo *R* ^2^ = 0.051) between eDNA probability of detection and filter membrane pore size (ranging from 0.2 to 5 μm) (Figure 5b). Adding random factors to the model, such as filter membrane type, did not result in a better explanation of the variation in *P*_eDNA_.

Spearman's correlation test indicated a significant positive correlation between filter membrane pore size, volume of water filtered (Spearman *ρ* = 0.12, *p* = .02), and the probability of detection.

**FIGURE 6:**
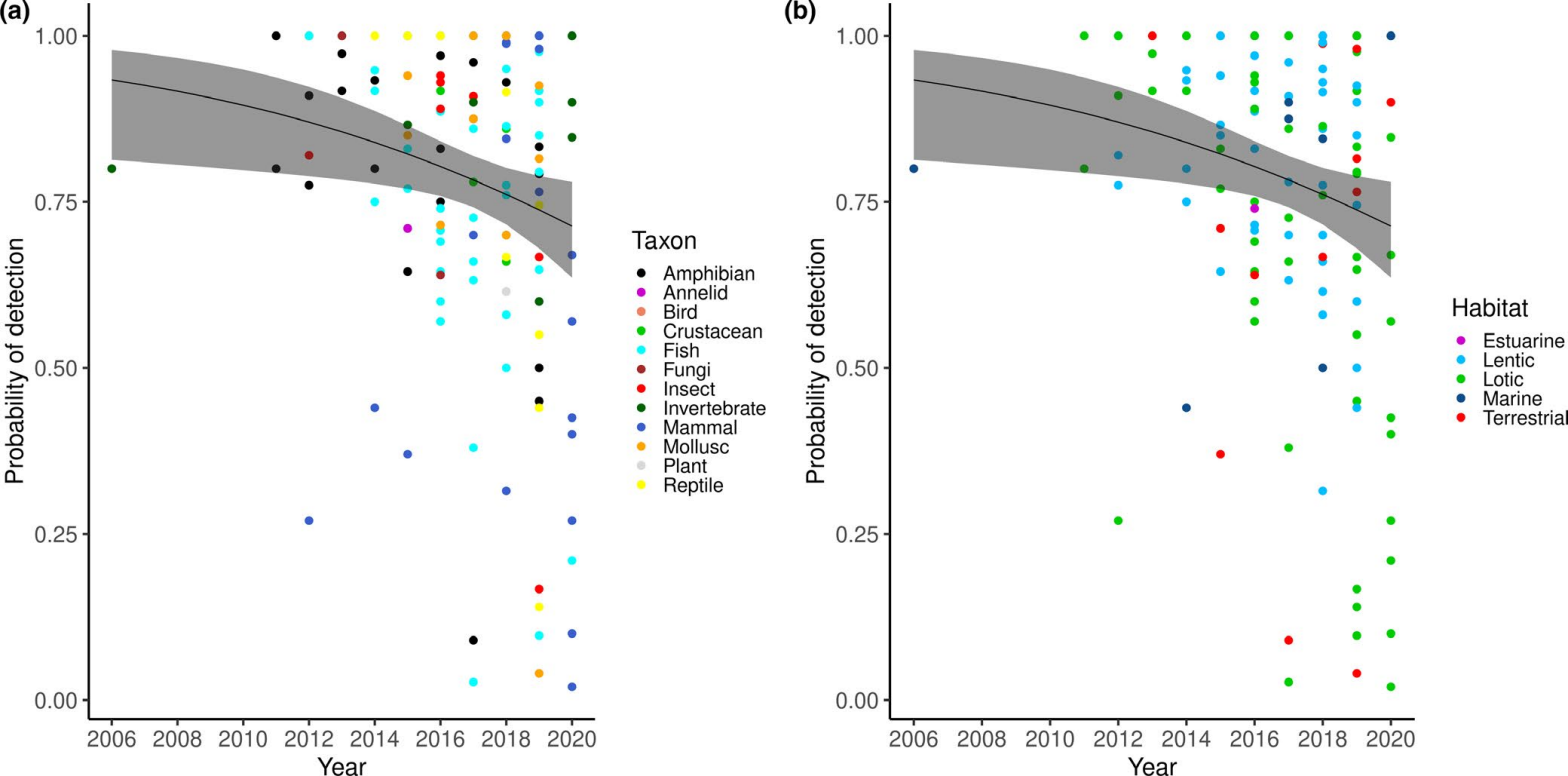
The time series model of *P*_eDNA_ for different taxa (a) and habitats (b). Model fit is plotted as a black line, with 95% confidence level as grey band

The time series modelling indicated that with time *P*_eDNA_ values decreased and this negative association was significant (*p* = .048, *n* = 115, Pseudo *R* ^2^ = 0.026). Adding habitats and taxa as explanatory factors did not significantly improve the model. Figure 6 shows that between 2015 and 2020 the number of studies reporting *P*_eDNA_ for terrestrial and freshwater lotic habitats increased. Similarly, studies reporting *P*_eDNA_ shifted from fish dominated to more taxonomically diverse, applying the eDNA method to the detection of reptiles, insects, and mammals.

#### 3.2.2 Analysis of categorical data

**FIGURE 7:**
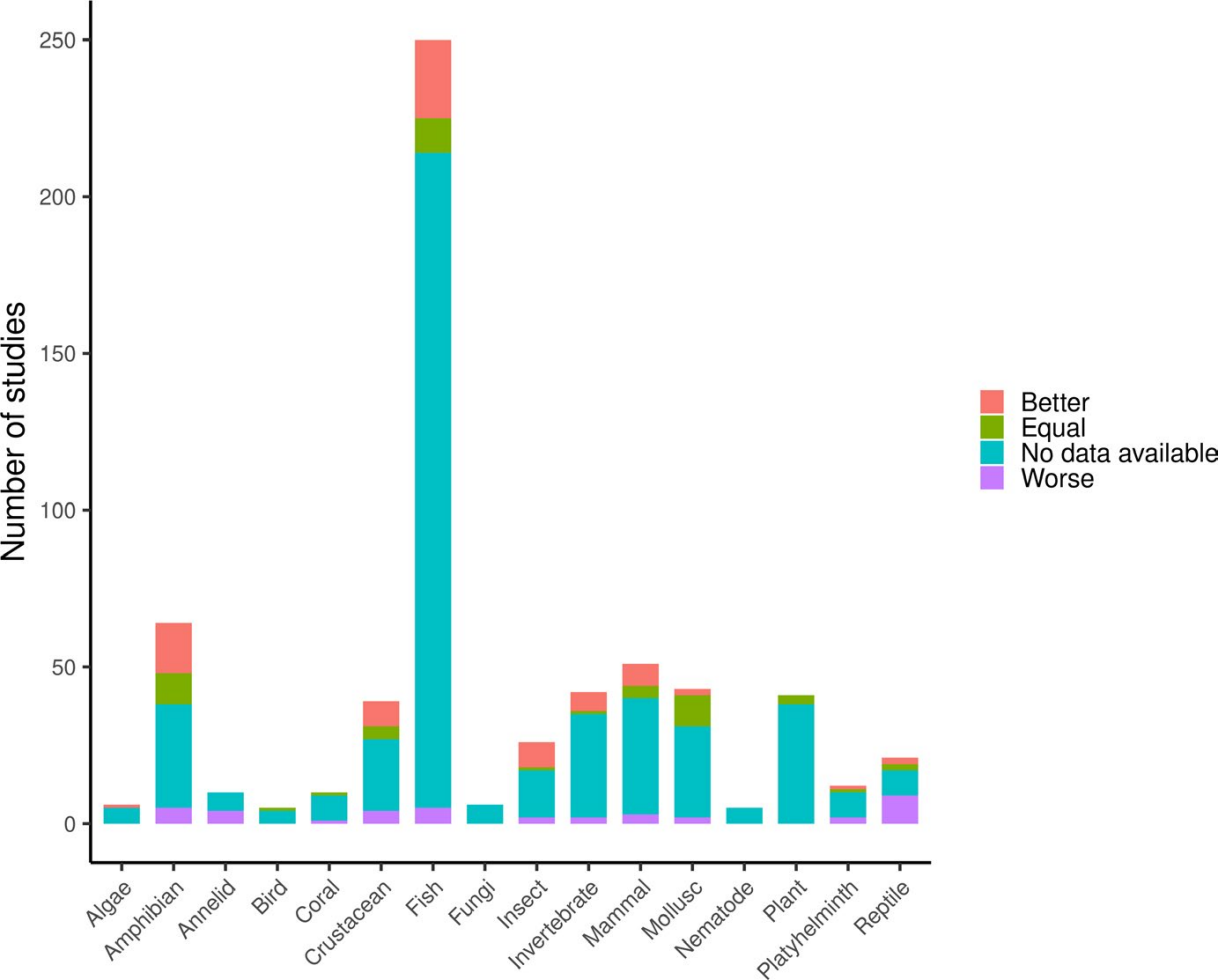
Number of studies that used eDNA method for different taxonomic groups and proportion of different comparison outcomes for each group, shown as stripes, where red signifies that eDNA outperformed traditional methods, green—method groups were equal, blue—traditional outperformed eDNA and grey—no comparison has been conducted

Due to insufficient sample sizes across other factors, only the effect of taxa, habitats, and sampling depth were investigated (Table S3). A *χ* ^2^ test detected a significant association between taxa and sensitivity (*χ* ^2^ = 58.17, *p* = .009, *n* = 153). Subsequent multiple correspondence analyses indicate that eDNA methods are (a) more sensitive than traditional methods for the detection of amphibians, insects, and invertebrates (such as tunicates, branchiopods, bryozoans, hydrozoans), (b) are on a par with traditional methods for mammals and molluscs, and (c) perform worse for reptiles and annelids (Figure S1). These conclusions mirror relative research effort (Figure 7), with amphibians and invertebrates among the most studied (67 and 74 studies, respectively) and reptiles and annelids among the least (22 and 10 studies, respectively).

## 4 DISCUSSION

This study indicates that eDNA outperforms traditional survey methods, but also highlights that broad statements about eDNA survey effectiveness in the literature are based on limited evidence. Just over one‐third of papers directly compare eDNA with traditional survey methods and only 9% give a quantitative measure of relative sensitivity. Comparisons of method sensitivity (170 papers), number of detectable species (75 papers), and cost‐effectiveness (19 papers) are made, with the latter being an underrepresented, but key consideration for conservation practitioners (Evans & Lamberti, 2018; McInerney & Rees, 2018; Qu & Stewart, 2017).

Our overall conclusion that environmental DNA is more sensitive, cheaper, and results in a higher number of detectable species may reflect a publication bias, as Beng and Corlett (2020) suggest that eDNA failures are less likely to be published. More recently (since 2018) there has been a decrease in *P*_eDNA_ values, which could be associated with the diversification of eDNA applications.

Here, comparisons of *P*_eDNA_ versus *P*_trad_, and the use of *P*_eDNA_ as a response variable in modeling and analysis of variance was hindered by the inconsistency in probability estimates. Authors either used occupancy models, calculated a ratio of positive and total observations or did not specify a method. Outcomes are variously described in terms of detection rate (Amberg et al., 2015; Biggs et al., 2015 ; Doi et al., 2017) or the probability of detection (Minamoto et al., 2017; Pilliod et al., 2013). Inconsistently described methods, terminology (Hunter et al., 2015), and results also limit the extent to which we could examine the factors influencing the outcome of comparative studies (Koricheva et al., 2013). Methodological information, in particular, lacked standardization, for example, sampling depth, filter membrane pore size, and whether qPCR was probe or SYBR‐based, despite the latter being important to report for qPCR studies (Bustin et al., 2009). The publication of Goldberg *et al*. in 2016 provided guidelines for reporting eDNA studies, and it would be interesting to track the progress of this in future meta‐analyses.

Results from our study support the view that qPCR results in a significantly higher *P*_eDNA_ than PCR (Amberg et al., 2015; Fernandez et al., 2018; Piggott, 2017; Thomsen et al., 2012; Turner et al., 2014; Wilcox et al., 2013; Williams et al., 2017). Quantitative PCR is more sensitive to low concentrations of eDNA in environmental samples, and samples that have been diluted to decrease amplification inhibition (Turner et al., 2014; Williams et al., 2017). Novel eDNA amplification methods, such as droplet digital PCR (ddPCR) and loop‐mediated isothermal amplification (LAMP), did not have a sufficient sample size for the purpose of our analysis. However, ddPCR has been shown to perform better than qPCR by Doi, Takahara, et al. (2015), Doi, Uchii, et al. (2015), Hamaguchi et al. (2018), Uthicke et al. (2018), and Brys et al. (2020) and is likely to become a popular method for future eDNA‐based surveys.

Although a significant difference in *P*_eDNA_ was detected for different environmental samples, we were not able to draw reliable conclusions about which related to the highest *P*_eDNA_. Only water had a sufficient sample size, while sediment, snow, saliva, soil, and other sources were understudied. This mirrors Jiang and Yang's (2017) conclusion that eDNA research has primarily focused on detecting species in aquatic environments. Genetic material can disperse due to water polarity and movement (Jeunen, Knapp, Spencer, Lamare, et al., 2019; Jeunen, Knapp, Spencer, Taylor, et al., 2019). Sediment and soil samples typically have more humic substances than water samples, and this might result in increased amplification inhibition (Buxton et al., 2017).

For samples of water, *P*_eDNA_ was positively associated with filter membrane pore size from 0.2 to 5 μm, however, the number of studies using pores larger than 3 μm was low. Smaller pore membranes can become clogged by organic matter and debris, limiting the volume of water that can be sampled. This issue has been reported several times (Franklin et al., 2018; Robson et al., 2016; Turner et al., 2014) and may account for the positive association between pore size and eDNA sensitivity. Prefiltering using larger pores has been suggested as a possible solution (Djurhuus et al., 2018; Li et al., 2018). In contrast, Turner et al. (2014), Robson et al. (2016), Kamoroff and Goldberg (2018) and Jeunen, Knapp, Spencer, Lamare, et al. (2019), Jeunen, Knapp, Spencer, Taylor, et al. (2019) detect a negative association between *P*_eDNA_ and membrane pore sizes, presumably due to the most abundant particles of eDNA being less than 0.2 μm (Turner et al., 2014). The relationship between pore size, water volume, and eDNA sensitivity continues to vary from case‐to‐case and sampling protocols should, therefore, be informed by the results of pilot studies (Goldberg et al., 2016; Harper, Buxton, et al., 2019; Harper, Griffiths, et al., 2019).

The results of *χ* ^2^ test of independence and multiple correspondence analysis indicate that eDNA methods are more sensitive for invertebrates and amphibians, perform worse for reptiles and annelids, and are as good for mammals and molluscs. This could be due to different eDNA shedding rates (Sansom & Sassoubre, 2017), different habitat types affecting eDNA dispersal (Andersen et al., 2012), or uneven research effort for these taxa, with amphibians studied more than mammals and reptiles.

Our study did not include all possible comparison criteria, such as how accurately abundance is estimated by eDNA and traditional methods (Buxton et al., 2017; Yates et al., 2019), how spatial scale coverage and sampling effort differ, and whether eDNA has a better carbon footprint. The effect of environmental factors such as season (Matsuhashi et al., 2019; McGee & Eaton, 2015) and physicochemical properties of habitat, can all contribute to eDNA degradation (Buxton et al., 2017) or increase eDNA shedding rates (Goldberg et al., 2018) and remain to be investigated in meta‐analyses. The recent reporting by Jeunen et al. (2020), indicating that primers used in parallel can improve eDNA performance will be an interesting factor to consider for future meta‐analyses of comparative studies.

To conclude, we recommend that further studies: (a) are more explicit with regard to comparison criteria, specifically what aspects of the eDNA and traditional methods are being compared; (b) provide quantitative evidence for all methods compared, for example, cost, number of detectable species, carbon footprint and probability of detection; (c) describe how quantitative measures were derived; and (d) for quantitative results, such as probability of detection or eDNA quantity, indicate sample size, measures of spread, for example, range, and units. Not all studies we looked at met these recommendations. We also recommend that eDNA community continue to test underrepresented amplification methods, such as ddPCR and LAMP, use diverse sample types such as sediment, snow, saliva, soil, or iDNA, and apply eDNA methods to a wide range of taxa, such as mammals, birds, reptiles, corals, plants, and fungi.

## CONFLICT OF INTEREST

The authors declare that they have no conflicts of interest to report.

## AUTHOR CONTRIBUTION

**Julija Fediajevaite:** Formal analysis (lead); Writing‐original draft (lead). **Victoria Priestley:** Conceptualization (equal); Writing‐review & editing (equal). **Richard Arnold:** Writing‐review & editing (equal). **Vincent Savolainen:** Funding acquisition (lead); Supervision (lead); Writing‐review & editing (equal).

## ETHICS STATEMENT

None relevant.

## Supporting information

Supplementary Material

## Data Availability

The data collected in this study are publicly available via Zenodo: http://doi.org/10.5281/zenodo.4465165 (metadata in Table S4).
