# Supplementary material for: Meta‐analysis shows that environmental DNA outperforms traditional surveys, but warrants better reporting standards
Source: Ecol Evol. 2021 Mar 18;11(9):4803–15. doi: 10.1002/ece3.7382 (PMC8093654; doi:10.1002/ece3.7382)
Supplement: Supplementary file 1 — Supplementary Material [file ECE3-11-4803-s001.docx]

**Supporting Information**

To estimate if the model was under- or over- dispersed, the dispersion statistic was calculated as in Zuur *et al.* (2013):

$Dispersion statistic= \frac{\chi^{2}}{residual degrees of freedom}$ (1)

If the model was found to be under- (dispersion statistic < 1) or over- dispersed (dispersion statistic > 1), the regression model was adjusted using the quasibinomial (link = "logit") family, which assumed the quasi-binomial error distribution.

$Pseudo R^{2}=\frac{null deviance-residual deviance}{residual deviance}$ (2)

Table S1. Key data on methodological information, extracted from the papers analyzed in this study.

| **Information on methods collected** | **Explanation** |
| --- | --- |
| Sampling depth | - At surface (if authors state “just below the surface” or “surface water was sampled”) - At depth (if authors state “just above the sediment” or e.g. “at 50 m depth”) - Both (if sampling occurred both at surface and at depth) |
| Capture method | - Precipitation (usually referred to as “ethanol and sodium acetate precipitation, abbreviated as “EP”) - Filtering (according to the filter membrane types:  1. Cellulose acetate membrane (CA) 2. Glass fibre membrane (GFF) 3. Mixed cellulose esters membrane (MCE) 4. Nitrocellulose membrane (NC) 5. Nylon mebrane 6. Polycarbonate membrane (PC) 7. Polycarbonate track-etched membrane (PCTE) 8. Polyethersulfone membrane (PES) 9. Polyvinylidene difluoride membrane (PVDF)) |
| Filter membrane pore size | The size of filter membrane pores (µm) |
| Volume of water filtered | The amount of water (L) passed through the filter membrane. |
| Source of eDNA sampled | The media containing eDNA that was sampled (e.g. water or soil). |
| DNA extraction method | Method of extracting DNA from the environmental media sampled. |
| DNA amplification method | - Polymerase chain reaction (PCR):  1. Conventional PCR (PCR) 2. Real-time quantitative PCR (qPCR) 3. Digital droplet PCR (ddPCR)  - Isothermal amplification (usually referred to as loop-mediated isothermal amplification (LAMP)) |
| Amplification markers | Target DNA or RNA regions for amplification |
| Sequencing method | Method of sequencing amplified DNA (e.g. Sanger sequencing or Illumina Miseq sequencing) |

Table S2. Kruskal-Wallis analysis of variance and modelling results, suggesting a significant difference among *P_eDNA_* values obtained by different amplification methods and sources sampled, as well as significant association with varying filter membrane pore sizes (significant results highlighted in red).

| **Test** | **Factor** | **Effect on *P_eDNA_*** | | |
| --- | --- | --- | --- | --- |
|  |  | **𝜒^2^ value** | **p value** | **Sample size** |
| Kruskal-Wallis analysis of variance | Taxa | 16.81 | 0.33 | 170 |
|  | Habitat | 2.83 | 0.59 | 152 |
|  | **Amplification** | **11.74** | **0.002** | **150** |
|  | Extraction | 9.71 | 0.29 | 153 |
|  | Capture | 10.05 | 0.35 | 129 |
|  | Markers | 20.68 | 0.19 | 122 |
|  | Depth | 0.81 | 0.67 | 55 |
|  | **Source** | **14.45** | **0.04** | **152** |
| Modelling | **Pore size** | NA | **0.03** | **117** |
|  | Volume | NA | 0.08 | 91 |

Table S3. 𝜒^2^ test of independence results, suggesting a significant association between different taxa studied and *P_eDNA_* (significant results highlighted in red). NA was assigned if the test could not be performed.

| **Test** | **Factor** | **Sensitivity** | | | **Cost-effectiveness** | | | **OTUs detected** | | |
| --- | --- | --- | --- | --- | --- | --- | --- | --- | --- | --- |
|  |  | **𝜒^2^ value** | **p value** | **Sample size** | **𝜒^2^ value** | **p value** | **Sample size** | **𝜒^2^ value** | **p value** | **Sample size** |
| 𝜒^2^ test | **Taxa** | **58.17** | **0.009** | **153** | NA | NA | 21 | NA | NA | 23 |
|  | Habitat | 6.49 | 0.59 | 153 | NA | NA | 21 | NA | NA | 65 |
|  | Amplification | NA | NA | 144 | NA | NA | 19 | NA | NA | 58 |
|  | Extraction | NA | NA | 143 | NA | NA | 16 | NA | NA | 64 |
|  | Capture | NA | NA | 155 | NA | NA | 18 | NA | NA | 61 |
|  | Markers | NA | NA | 140 | NA | NA | 20 | NA | NA | 79 |
|  | Depth | 1.48 | 0.83 | 57 | NA | NA | 9 | 3.64 | 0.46 | 24 |
|  | Source | NA | NA | 147 | NA | NA | 17 | NA | NA | 62 |
|  | Sequencing | - | - | - | NA | NA | 11 | NA | NA | 50 |


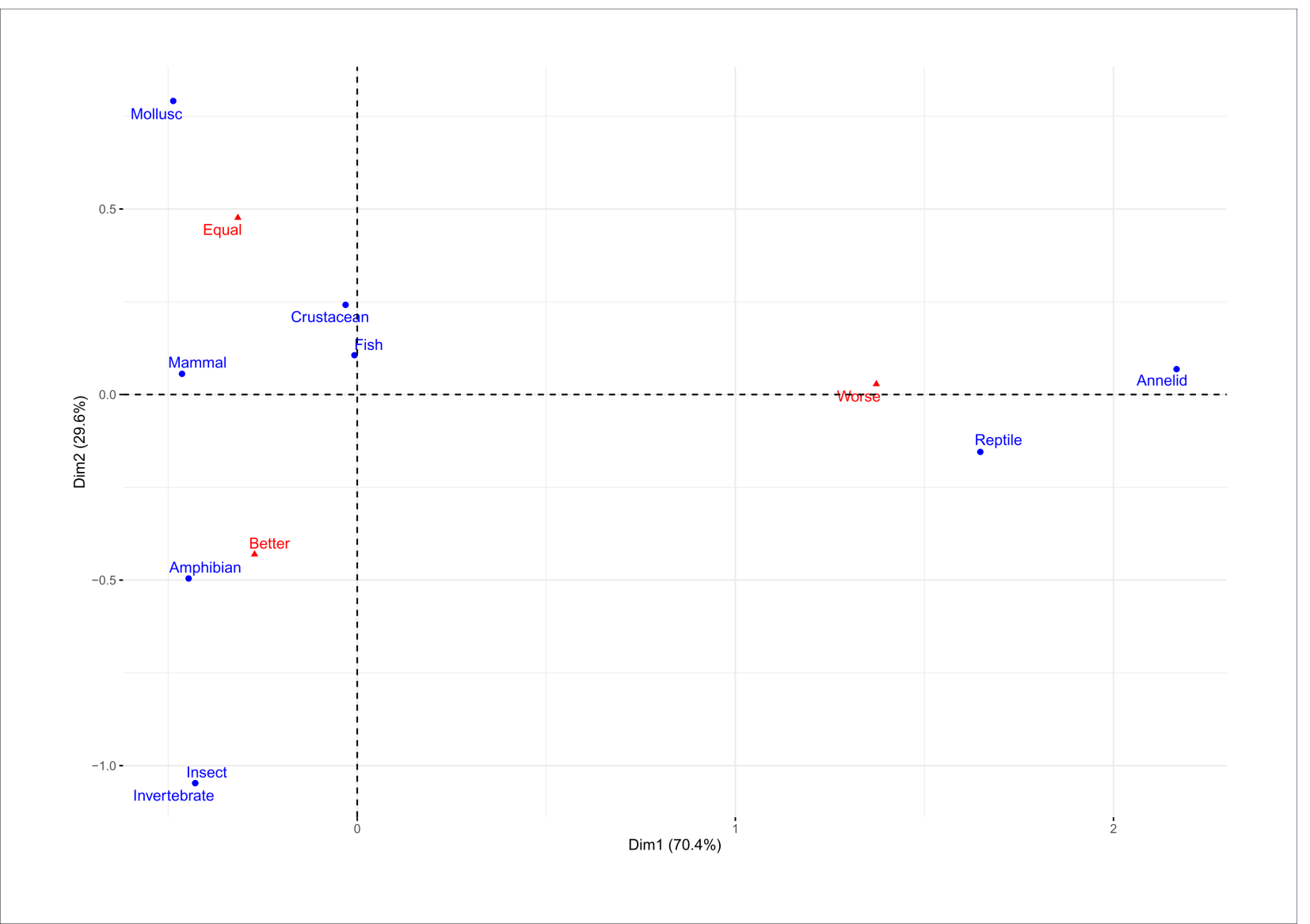


Fig. S1. Multiple correspondence analysis visualisation of a global pattern within the data. Taxa are represented by points and comparison outcome categories by triangles. The distance between any taxa or comparison outcome category gives a measure of their similarity. Points or triangles with similar profile are situated in vicinity to each other on this factor map. Dimension 1 described 70.4 % of variation and Dimension 2 described 29.6 % variation in data.

Table S4. Description of publicly available data, that consists of 11 worksheets.

| Title of worksheet | Description | Names of variables |
| --- | --- | --- |
| Raw | The primary compilation of data retrieved from each paper’s methods, results, discussion sections and/or supporting information. Data in this worksheet is not standardised and one entry can have multiple values. | Title of paper, Authors, Species studied, Broader taxonomic unit, Sampling technique, Capture technique, Extraction method, Amplification method, Sequencing method, Time/season, Habitat, Broader habitat type, Location, Source of eDNA, Probability of detection, eDNA vs traditional conclusion given by authors, eDNA vs trad rating assigned (1-eDNA better, 0-equal, -1-worse than trad, u-unclear), eDNA yield, LOD and LOQ, Remarks. |
| Standardised | Consists of raw data and standardised entries, showing the process of assigning specific codes and unique values for each entry. Variables “Sensitivity wise”, “Cost wise” and “OTUs wise” signify comparison outcomes as derived from the variable “eDNA vs traditional conclusion given by authors” in the Raw data worksheet. | Species studied, Broader taxonomic unit, Sampling technique, Capture technique, Capture_standardised, Pore size, Volume filtered, Extraction method, Extraction_standardised, Amplification, Markers, Sequencing method, Time/season, Year, Habitat, Broader habitat type, Location, Country, Source of eDNA, Probability of detection, eDNA prob of detect, trad prob of detect, eDNA vs traditional conclusion given by authors, Sensitivity wise, Cost wise, OTUs wise, eDNA yield, Max Yield (ng/ul), LOD (copies/reaction), LOQ, LOD and LOQ. |
| Habitats | Subset of Standardised data, having entries associated with habitats. If a paper studied multiple habitats, all of them were included as separate rows in this data frame. | Habitat, Habitat Code, eDNA prob of detect, Sensitivity wise, Cost wise, OTUs wise, Yield. |
| Taxa | Subset of Standardised data, having entries associated with taxa. If a paper studied multiple taxa, all of them were included as separate rows in this data frame. | Taxon, Markers, eDNA prob of detect, Sensitivity wise, Cost wise, OTUs wise, Yield, LOD, LOQ. |
| Fiters | Subset of Standardised data, having entries associated with capture techniques. If a paper used multiple capture techniques, all of them were included as separate rows in this data frame. | Capture, Pore size, Volume filtered, Year, Country, eDNA prob of detect, Sensitivity wise, Cost wise, OTUs wise, Yield. |
| Extractions | Subset of Standardised data, having entries associated with extraction techniques. If a paper used multiple extraction techniques, all of them were included as separate rows in this data frame. | Extraction, eDNA prob of detect, Sensitivity wise, Cost wise, OTUs wise, Yield. |
| Sampling | Subset of Standardised data, having entries associated with sampling techniques. If a paper used multiple sampling techniques, all of them were included as separate rows in this data frame. | Sampling, eDNA prob of detect, Sensitivity wise, Cost wise, OTUs wise, Yield. |
| Amplification | Subset of Standardised data, having entries associated with amplification techniques. If a paper used multiple amplification techniques, all of them were included as separate rows in this data frame. | Amplification, Year, eDNA prob of detect, Sensitivity wise, Cost wise, OTUs wise, Yield, LOD, LOQ. |
| Sequencing | Subset of Standardised data, having entries associated with sequencing techniques. If a paper used multiple sequencing techniques, all of them were included as separate rows in this data frame. | Sequencing method, Year, eDNA prob of detect, Sensitivity wise, Cost wise, OTUs wise, Yield. |
| Markers | Subset of Standardised data, having entries associated with markers. If a paper used multiple markers, all of them were included as separate rows in this data frame. | Markers, eDNA prob of detect, Sensitivity wise, Cost wise, OTUs wise, Yield, LOD, LOQ. |
| Source | Subset of Standardised data, having entries associated with eDNA source. If a paper used multiple sources, all of them were included as separate rows in this data frame. | Source of eDNA, eDNA prob of detect, Sensitivity wise, Cost wise, OTUs wise, Yield. |
